# Relationship between executive function and dual-task walking in people with Parkinson's disease

**DOI:** 10.3389/fnagi.2025.1585524

**Published:** 2025-06-18

**Authors:** Jun-Hong Zhou, Ray-Yau Wang, Yo-Tsen Liu, Shih-Jung Cheng, Hsin-Hsuan Liu, Yea-Ru Yang

**Affiliations:** ^1^Department of Physical Therapy and Assistive Technology, National Yang Ming Chiao Tung University, Taipei, Taiwan; ^2^Division of Epilepsy, Neurological Institute, Taipei Veterans General Hospital, Taipei, Taiwan; ^3^School of Medicine, National Yang Ming Chiao Tung University, Taipei, Taiwan; ^4^Institute of Brain Science, School of Medicine, National Yang Ming Chiao Tung University, Taipei, Taiwan; ^5^Brain Research Center, National Yang Ming Chiao Tung University, Taipei, Taiwan; ^6^Department of Neurology, MacKay Memorial Hospital, Taipei, Taiwan; ^7^Department of Physical Therapy, Chung Shan Medical University, Taichung, Taiwan

**Keywords:** cognition, concurrent task, inhibitory control, memory, movement disorders

## Abstract

**Background:**

Cognitive impairment may present early in people with Parkinson's disease (PwPD), with deficits in executive function potentially impacting gait performance. Previous studies have investigated the association between dual-task walking and executive function in PwPD; however, the results were inconsistent, and the correlation between dual-task walking and subdomains of executive function has not been explored. This study aims to examine the correlation between dual-task walking and subdomains of executive function in PD and assess the predictive power of different subdomains of executive function on dual-task walking performance.

**Methods:**

This cross-sectional study included 30 PwPD. Gait was assessed under single-task walking, cognitive dual-task walking, and motor dual-task walking conditions. Executive function was evaluated using the Trail Making Test (TMT), Stroop Color and Word Test (SCWT), and Digit Span Test (DST). Correlation analyses (Pearson or Spearman, as appropriate) and linear regression analyses were used to examine the contribution of executive function subdomains to gait variables that showed significant correlations.

**Results:**

Walking speeds under both dual-task conditions were moderately correlated with performance on the TMT Part A and the SCWT. In contrast, stride length during dual-task walking showed broader associations, demonstrating significant correlations with multiple executive function measures. Stepwise linear regression analysis revealed that the SCWT was the only significant predictor of walking speed under both dual-task conditions. For stride length during cognitive dual-task walking, the SCWT remained a significant predictor, while in the motor dual-task condition, both the SCWT and the Forward DST contributed significantly. Specifically, two regression models were significant for stride length during motor dual-task walking: Model 1 included only the SCWT, while Model 2 incorporated both the SCWT and Forward DST. Among dual-task cost outcomes, only the cost of stride length during cognitive dual-task walking was significantly correlated with TMT Part A; however, this association did not remain significant in subsequent regression analyses.

**Conclusion:**

This study indicates that, among various executive function assessments, the SCWT shows the strongest correlation with dual-task gait performance in PwPD. This suggests that inhibitory control plays a key role in regulating dual-task walking in individuals with PD.

## 1 Introduction

Movement disorders are among the primary concerns for people with Parkinson's disease (PwPD), significantly impairing gait by reducing walking speed, stride length, and step length while increasing gait variability (Morris et al., 1996; Hausdorff et al., 1998; Roemmich et al., 2012). Beyond movement disorders, ~25–80% of PwPD also experience comorbid cognitive impairment (Aarsland et al., 2003; Fang et al., 2020; Aarsland et al., 2021). These cognitive deficits include executive dysfunction, which may emerge even in the early stages of PD (Dirnberger and Jahanshahi, 2013). Executive function refers to a set of cognitive processes that regulate goal-directed behavior, from goal formulation and attentional control to task execution and outcome achievement (Dirnberger and Jahanshahi, 2013). Cognitive impairment or executive dysfunction may be associated with poorer walking performance in PwPD (Shearin et al., 2021; Kang et al., 2022).

Walking in daily life often requires performing concurrent tasks, resulting in dual-task walking. Dual-task walking refers to the act of walking while simultaneously performing an additional task, which can be either cognitive (e.g., talking, thinking, or solving problems) or motor (e.g., carrying an object or manipulating something with the hands). This process places demands on executive function, which involves attention allocation, task switching, and the coordination of multiple tasks, and is critical for managing the increased cognitive load during dual-task situations (Szameitat and Students, 2022). For PwPD, dual-task walking is particularly challenging (Kelly et al., 2012; Raffegeau et al., 2019; Johansson et al., 2021), likely due to impairments in executive function (Rochester et al., 2008).

Although it has been proposed that executive function contributes to the modulation of dual-task walking, existing evidence remains limited and inconsistent. In assessing dual-task walking, researchers commonly evaluate spatiotemporal gait parameters (such as speed, stride length, and cadence) or compute the dual-task cost, which quantifies the relative change in performance compared to single-task walking. A previous study reported a significant correlation between executive function (assessed using the Trail-Making Test) and walking features under cognitive dual-task conditions (Piet et al., 2024). In contrast, another study found no significant correlation between executive function (measured by the Brixton test) and walking speed during motor dual-task walking (Rochester et al., 2008). Regarding the relationship between executive function and dual-task cost on walking, two studies identified a significant link between executive function (measured using the Brixton test) and the dual-task cost on walking speed in motor dual-task walking (Rochester et al., 2008; Lord et al., 2010). Similarly, another study reported a significant correlation between cognitive status (assessed using a battery covering executive function, episodic memory, and visuospatial skills) and the dual-task cost on walking speed during cognitive dual-task walking (Johansson et al., 2021). In contrast, Stegemöller et al. (2014) found no significant correlation between executive function (represented by a composite score derived from the Stroop XXX, 2-back, visual working memory, and digit symbol substitution tests) and the dual-task cost on walking speed or step length during cognitive dual-task walking. Likewise, Fernández-Lago et al. (2024) found no significant association between overall executive function performance (based on a composite score from the Trail Making Test Part B, Digit Span Backwards, Tower of London, Word Fluency, Corsi Block Test, and Berg Card Sorting Test) and the dual-task cost on walking speed or stride length during cognitive dual-task walking. However, they did observe a significant correlation between executive function performance and the dual-task cost on cadence during cognitive dual-task walking (Fernández-Lago et al., 2024). These discrepancies may arise from methodological and conceptual differences across studies, including variations in dual-task conditions (e.g., cognitive vs. motor tasks with differing complexity) and the use of heterogeneous executive function assessment tools. Notably, many studies treat executive function as a unitary construct, rather than distinguishing among its subdomains, which may obscure specific relationships with dual-task walking performance.

Executive function includes core domains such as inhibitory control, working memory, and cognitive flexibility (Diamond, 2013), and the influence of each subdomain on dual-task walking performance may differ. For instance, it has been suggested that inhibitory control tasks interfere more with dual-task walking than working memory tasks (St George et al., 2022). Further investigation is warranted to explore how distinct executive function subdomains relate to dual-task walking performance. A clearer understanding of these relationships could inform the development of targeted cognitive and motor interventions aimed at improving gait safety and functional mobility in PwPD, particularly in situations that require multitasking in daily life. Therefore, the present study aims to investigate the relationship between specific executive function subdomains and two types of dual-task walking in PwPD, and to further analyze the extent to which each subdomain influences performance under these dual-task walking conditions.

## 2 Methods

### 2.1 Participants

This study enrolled a total of 30 participants referred by neurologists. All participants were required to meet the clinical diagnostic criteria for idiopathic Parkinson's disease established by the United Kingdom Parkinson's Disease Society Brain Bank (Gibb and Lees, 1988). The inclusion criteria were as follows: (1) Hoehn-Yahr stage I to III; (2) age not exceeding 80 years; (3) ability to walk 10 meters independently without the use of assistive devices; (4) a Mini-Mental State Examination score of at least 24; and (5) stable medication regimen. The exclusion criteria were as follows: (1) the presence of any additional disorders or comorbidities, other than Parkinson's disease, that could potentially impact the study; (2) any neurological or musculoskeletal conditions that might interfere with the study procedures or influence the interpretation of the results; and (3) severe gait freezing, as indicated by a score >15 on the Freezing of Gait Questionnaire (Tao et al., 2021).

### 2.2 Study design and procedure

This study employed a cross-sectional design wherein all participants underwent a single assessment session. The evaluation encompassed gait performance, executive function, and global cognitive ability. All assessments were conducted while participants were in the ON medication state. Before the commencement of the study, the research protocol was approved by the Institutional Review Board. All participants were fully informed about the study details and provided written informed consent before participating.

### 2.3 Outcome measure

#### 2.3.1 Measures of gait parameters

Gait was assessed using the GAITRite walkway system (CIR Systems, Inc., Havertown, Pennsylvania), a portable electronic walkway embedded with pressure sensors, 488 cm in length and 61 cm in width, that capture both temporal and spatial gait parameters with strong concurrent validity and reliability (Bilney et al., 2003). Gait parameters were assessed under three walking conditions: single-task, cognitive dual-task, and motor dual-task. Each condition involved six trials of self-selected speed walking, with each trial covering a distance of ~6 m, resulting in a total of 18 recorded walking trials across all conditions. To mitigate order effects, the sequence of the three conditions was randomized. The recorded gait parameters included walking speed (m/s) and stride length (m). During the cognitive dual-task walking condition, participants were required to perform a serial subtraction task (subtracting by threes sequentially) starting from a randomly selected three-digit number >600 while walking (Plotnik et al., 2011; Stegemöller et al., 2014). This approach was intended to minimize potential learning, rehearsal, or memorization effects across the six dual-task walking trials. In the motor dual-task walking condition, participants were asked to walk while carrying a cup filled to 80% of its capacity with water, ensuring no water was spilled during the task. The dual-task cost was calculated to quantify the interference caused by the dual-task conditions relative to single-task walking. The dual-task cost was computed using the following formula (illustrated here for walking speed; Kelly et al., 2010):

Dual-task cost for walking speed (%) = [(*Single*-*task walking speed* – *Dual*-*task walking speed*)/*Single*-*task walking speed*] × 100%

This metric was applied to both walking speed and stride length to evaluate the impact of dual-task interference on gait performance.

#### 2.3.2 Measures of executive function

The assessment of executive function encompasses the Trail Making Test, the Stroop Color and Word Test, and the Digit Span Test. The Trail Making Test is a simple assessment tool to evaluate visuoperceptual ability, attention, task-switching capacity, and cognitive flexibility (Kortte et al., 2002; Lai et al., 2020). This test is divided into parts A and B. Part A consists of numbers 1–25, each enclosed in a circle. Participants are instructed to connect the numbers sequentially as quickly as possible. A shorter completion time indicates better visuoperceptual performance and attention. Part B consists of 12 encircled numbers (1–12) and 12 encircled letters (A–L). Participants are required to alternately connect the numbers and letters (1–A−2–B−3–C…−12–L). A shorter completion time indicates better visuoperceptual performance, divided attention, and set-shifting ability.

The Stroop Color and Word Test assesses the ability to inhibit cognitive interference (Scarpina and Tagini, 2017). The test employed the Stroop color and word incongruence paradigm, wherein four Chinese color characters (black, blue, red, and yellow) were randomly printed in four different colors (Wang et al., 2018). Participants were required to rapidly articulate the printed color of each character within a 45-s time frame. The examiner recorded the number of correct responses, with a higher count indicating superior inhibitory control.

The Digit Span Test is a simple assessment designed to measure short-term memory and working memory (Richardson, 2007; Hilbert et al., 2015). This test comprises forward and backward recall components (Lezak et al., 2012; Guo et al., 2024). The forward digit span test primarily assesses short-term memory, while the backward digit span test focuses on evaluating working memory. During the test, participants are required to repeat the numbers read aloud by the examiner (in the same order for the forward test and reverse order for the backward test). The total score for the forward digit span test is 16 points, and for the backward digit span test, 14 points. A higher number of correctly repeated digits indicates better cognitive performance.

### 2.4 Sample size

The sample size estimation was conducted using G^*^Power 3.1. The “Linear multiple regression: Fixed model, single regression coefficient” option was selected. The effect size (*f*^2^ = 0.28) was derived from the correlation (*r*) between cognitive dual-task walking and the Trail Making Test reported in a previous study (Piet et al., 2024). Assuming an alpha level of 0.05, a statistical power of 0.80, and three executive function subdomains as predictors, the analysis indicated that a minimum of 30 participants was required.

### 2.5 Statistical analysis

Data analysis was conducted using SPSS version 24.0, with statistical significance set at *p* ≤ 0.05. Descriptive statistics were used to summarize demographic data, gait parameters under three walking conditions, global cognitive ability, and executive function. A one-way repeated measures analysis of variance (ANOVA) was performed to compare gait parameters between single-task and dual-task walking conditions. The normality of continuous variables was assessed using the Shapiro–Wilk test. Pearson correlation coefficients were calculated for variables with normal distributions, while Spearman correlation coefficients were used for non-normally distributed variables to examine the relationship between executive function and dual-task gait performance. For gait parameters that showed significant correlations with multiple subdomains of executive function, a stepwise linear regression analysis was performed to determine the unique contribution of each subdomain. When a gait parameter was significantly correlated with only one executive function subdomain, a simple linear regression analysis was conducted to assess the contribution of that specific independent variable.

## 3 Results

### 3.1 Demographic and clinical characteristics

The demographic and clinical characteristics of the participants are presented in Table 1, while gait parameters under the three walking conditions are summarized in Table 2. A one-way repeated measures ANOVA identified significant differences in walking speed (*p* < 0.001) and stride length (*p* < 0.001) across the three walking conditions. *Post-hoc* analysis indicated that walking speed and stride length were significantly different during both dual-task walking conditions compared to single-task walking (*p* < 0.001). However, no significant differences were found between the cognitive and motor dual-task walking conditions for either walking speed or stride length.

**Table 1:** Demographic and clinical characteristics.

**Characteristics**	**Mean ±SD (*n* = 30)**	**Range**
Age (years)	64.27 ± 7.39	42–73
Gender (male/female)	18/12	
Disease duration (years)	5.09 ± 4.62	0.2–20
Hoehn and Yahr stage (I–III)	1.82 ± 0.69	1.0–3.0
More affected side (left/right)	17/13	
Mini-mental state examination	28.93 ± 1.60	25–30
Education (years)	14.07 ± 4.44	3–23
Levodopa equivalent daily dosages (mg)	431.92 ± 311.95	5–1,299
Freezing of Gait Questionnaire	1.50 ± 1.85	0–6
MDS-UPDRS III	18.21 ± 14.09	0–58
Trail Making test—part A (s)	39.03 ± 24.74	14.20–116.01
Trail Making test—part B (s)	96.49 ± 68.09	30.86–300.00
Stroop Color and Word test (n)	31.90 ± 11.88	14–60
Digit Span test—forward (n)	12.77 ± 2.67	7–16
Digit Span test—backward (n)	7.07 ± 2.64	2–14

SD, standard deviation; MDS-UPDRS III, Movement Disorder Society–Unified Parkinson Disease Rating Scale motor score; s, second; *n*, number.

**Table 2:** Comparison of gait parameters across different walking conditions.

**Gait parameters**	**Single-task walking**	**Cognitive dual-task walking**	**Motor dual-task walking**	***p*-value**
Speed (m/s)	1.06 ± 0.20	0.92 ± 0.24^***^	0.91 ± 0.22^***^	< 0.001
Stride length (m)	1.12 ± 0.18	1.03 ± 0.22^***^	0.99 ± 0.20^***^	< 0.001
Dual-task cost of speed (%)	–	13.97 ± 11.43	14.57 ± 10.21	
Dual-task cost of stride length (%)	–	8.42 ± 10.54	11.92 ± 8.24	

^***^The comparison with single-task walking reached a significance level of *p* < 0.001.

### 3.2 Correlation between executive function and dual-task walking

Table 3 presents the correlations between executive function and dual-task walking performance. Gait speed during cognitive dual-task walking was significantly correlated with the Trail Making Test Part A (*r* = −0.427) and Stroop Color and Word Test (*r* = 0.453). Stride length during cognitive dual-task walking showed moderate correlations with the Trail Making Test Part A (*r* = −0.532), Trail Making Test Part B (*r* = −0.468), Stroop Color and Word Test (*r* = 0.515), and Digit Span Backward Test (*r* = 0.371). Similarly, gait speed during motor dual-task walking was significantly correlated with the Trail Making Test Part A (*r* = −0.466) and Stroop Color and Word Test (*r* = 0.470). Stride length during motor dual-task walking exhibited moderate correlations with all executive function tests (*r* = −0.592 to 0.554). The dual-task cost of stride length in cognitive dual-task walking showed a significant correlation with the Trail Making Test Part A (*r* = 0.375). However, the dual-task cost of gait speed in the cognitive dual-task condition and the dual-task cost of gait speed and stride length in the motor dual-task condition showed no significant correlations with executive function.

**Table 3:** Correlation between executive function and dual-task walking.

**Executive functions**	**Cognitive dual-task walking**				**Motor dual-task walking**			
	**Speed**	**Stride length**	**DTC of speed**	**DTC of stride length**	**Speed**	**Stride length**	**DTC of speed**	**DTC of stride length**
	***r***	***r***	***r***	***r***	***r***	***r***	***r***	***r***
Trail Making test—part A (s)	−0.427^*^	−0.532^*^	0.101	0.375^*^	−0.466^*^	−0.592^*^	0.176	0.316
Trail Making test—part B (s)	−0.261	−0.468^*^	0.032	0.301	−0.211	−0.432^*^	−0.041	0.220
Stroop Color and Word test (n)	0.453^*^	0.515^*^	−0.056	−0.237	0.470^*^	0.554^*^	−0.050	−0.249
Digit Span test—forward (n)	0.137	0.256	0.194	0.057	0.313	0.479^*^	−0.148	−0.318
Digit Span test—backward (n)	0.154	0.371^*^	0.187	0.028	0.176	0.406^*^	0.156	−0.081

DTC, dual-task cost; s, second; *n*, number.

^*^p < 0.05.

### 3.3 Influence of executive function on dual-task walking

**Figure 1:**
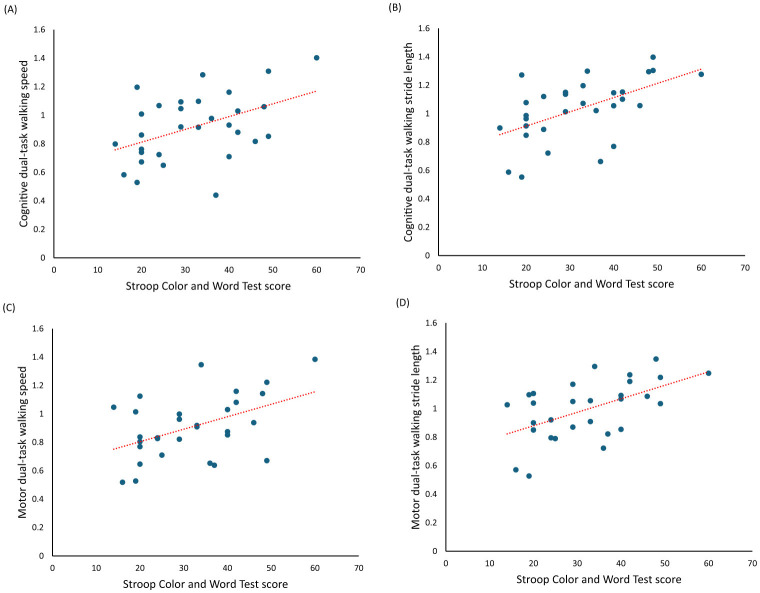
The scatterplot with the regression line. **(A)** Showed a relationship between cognitive dual-task walking speed and Stroop Color and Word test score. **(B)** Showed a relationship between cognitive dual-task walking stride length and Stroop Color and Word test scores. **(C)** Showed a relationship between motor dual-task walking speed and Stroop Color and Word test scores. **(D)** Showed a relationship between motor dual-task walking stride length and Stroop Color and Word test scores.

Table 4 presents the linear regression analysis of executive function and dual-task walking performance. The results of the stepwise linear regression analysis for cognitive dual-task walking speed revealed that the best model (adjusted *R*^2^ = 0.177, *F* = 7.228, *p* = 0.012) included only the Stroop Color and Word Test (β = 0.453, *t* = 2.688, *p* = 0.012; Figure 1A). For cognitive dual-task walking stride length, the stepwise linear regression analysis showed that the best model (adjusted *R*^2^ = 0.272, *F* = 11.834, *p* = 0.002) included only the Stroop Color and Word Test (β = 0.545, *t* = 3.440, *p* = 0.002; Figure 1B). Although the Trail Making Test Part A was significantly correlated with the dual-task cost of stride length during cognitive dual-task walking, it did not emerge as a significant predictor in the simple linear regression analysis.

**Table 4:** Linear regression analysis of executive function and dual-task walking.

**Walking condition**	**Results of the model**	**Adjusted *R*^2^**	**Significant factor**	**β**	***t***	***p***
**Cognitive dual-task walking**						
Speed^†^	*F*_(1, 29)_ = 7.228, *p* = 0.012	0.177	Stroop Color and Word test	0.453	2.688	0.012
Stride length^†^	*F*_(1, 29)_ = 11.834, *p* = 0.002	0.272	Stroop Color and Word test	0.545	3.440	0.002
DTC of stride length^‡^	*F*_(1, 29)_ = 0.844, *p* = 0.366	−0.005	Trail Making test—part A	0.171	0.919	0.366
**Motor dual-task walking**						
Speed^†^	*F*_(1, 29)_ = 7.943, *p* = 0.009	0.193	Stroop Color and Word test	0.470	2.818	0.009
Stride length^†^–Model 1	*F*_(1, 29)_ = 12.379, *p* = 0.002	0.282	Stroop Color and Word test	0.554	3.518	0.002
Stride length^†^–Model 2	*F*_(2, 29)_ = 11.143, *p* < 0.001	0.412	Stroop Color and Word test	0.428	2.850	0.008
			Digit Span test—forward	0.402	2.679	0.012

DTC, dual-task cost.

^‡^Simple linear regression analysis; ^†^stepwise linear regression analysis.

Regarding motor dual-task walking speed, the stepwise linear regression analysis indicated that the best model (adjusted *R*^2^ = 0.193, *F* = 7.943, *p* = 0.009) included only the Stroop Color and Word Test (β = 0.470, *t* = 2.818, *p* = 0.009; Figure 1C). For motor dual-task walking stride length, the stepwise linear regression analysis identified two optimal models: Model 1 (adjusted *R*^2^ = 0.282, *F* = 12.379, *p* = 0.002) included only the Stroop Color and Word Test (β = 0.554, *t* = 3.518, *p* = 0.002; Figure 1D), while Model 2 (adjusted *R*^2^ = 0.412, *F* = 11.143, *p* < 0.001) included both the Stroop Color and Word Test (β = 0.428, *t* = 2.850, *p* = 0.008) and the Digit Span Forward Test (β = 0.402, *t* = 2.679, *p* = 0.012).

## 4 Discussion

To our knowledge, this study is the first to analyze the impact of various subdomains of executive function on dual-task walking performance in PwPD. The findings indicate that dual-task walking speed is specifically related to inhibitory control within the subdomain of executive function, with stepwise linear regression analysis further confirming the contribution of inhibitory control to dual-task walking speed. Stride length during dual-task walking shows moderate correlations with nearly all subdomains of executive function. Stepwise linear regression analysis reveals that inhibitory control is the primary contributor to stride length during cognitive dual-task walking, while inhibitory control and/or short-term memory contribute to stride length during motor dual-task walking. These results highlight the significant role of inhibitory control in dual-task walking in PwPD.

A recent study revealed a significant association between global cognitive function (measured by the mini-mental state examination) and gait speed during cognitive dual-task walking in PwPD (Ivaniski-Mello et al., 2023). This finding highlights the potential impact of cognitive impairment on motor performance, particularly in complex tasks that require dual-tasking. PwPD often exhibit limited attentional resources (Yogev et al., 2005), which makes them more susceptible to interference during dual-task performance, resulting in reduced gait speed. When performing dual tasks, individuals are required to allocate attention and resources to simultaneously manage the primary task (walking) and the secondary task (cognitive or motor). The impaired executive function may lead to insufficient resource allocation, thereby affecting walking performance. Therefore, it is necessary to further elucidate the correlation between executive function and dual-task walking performance in PwPD. Our findings highlight the significant role of inhibitory control in dual-task walking performance. To date, insufficient literature explores the mechanisms linking inhibitory control to dual-task walking performance. Although direct evidence is lacking, we hypothesize two potential mechanisms through which inhibitory control may contribute most significantly to dual-task walking. First, during dual-task walking, superior inhibitory control may mitigate the interference of the secondary task on walking, thereby preserving the maximal degree of automaticity in walking. Second, PwPD may experience motor blocks when attempting to perform alternating upper limb movements (Vercruysse et al., 2012, 2014). The specific task of generating alternating movements relies on inhibitory control, as achieving regular alternating movements requires coordinated interhemispheric inhibition (Hummel et al., 2002; Daffertshofer et al., 2005). Therefore, we hypothesize that inhibitory control plays a crucial regulatory role in executing alternating movements during walking. Additionally, a study demonstrated that inhibitory control is the most robust predictor of stepping errors during the gait adaptability test (Caetano et al., 2019). Taken together, these findings suggest that PwPD with superior inhibitory control exhibit enhanced gait adaptability, enabling them to more effectively modulate their gait in response to external environmental challenges or secondary task interference.

In addition to inhibitory control, our findings indicate that the optimal model for predicting stride length during motor dual-task walking includes short-term memory as a significant predictor. Consistent with our findings, Killane et al. (2014) found that poorer short-term memory and slower processing speed were the most significant cognitive contributors to slower gait speed in dual-task walking among community-dwelling older adults. The neural mechanisms linking short-term memory to dual-task walking are not yet fully understood. However, cerebral imaging and behavioral studies suggest that short-term memory and dual-task walking share common neural pathways (Nee and Jonides, 2008; Pizzamiglio et al., 2017). Short-term memory plays a crucial role in motor dual-task walking by allowing individuals to integrate cognitive processing with motor actions in real time. This function is particularly important in conditions like PD, where both motor function and memory may be impaired.

Although significant correlations have been observed between executive function and dual-task walking performance, the present study did not find significant associations between dual-task cost and specific subdomains of executive function. Previous research has reported inconsistent correlations between dual-task cost and executive function (Rochester et al., 2008; Lord et al., 2010; Stegemöller et al., 2014; Johansson et al., 2021), which may be due to several factors, including the difficulty and nature of the secondary task, evaluation methods, medication status, and individual participant capabilities. Further research is needed to clarify the influence of executive function on dual-task cost.

This study has several limitations. First, the sample size was relatively small (*n* = 30), which may limit the generalizability of the findings. Although the sample size met the requirements for statistical power, a larger sample could reduce estimation error, increase the robustness of the results, and enhance both representativeness and external validity. Second, the study did not apply Bonferroni correction due to the limited sample size, which inherently reduces statistical power. Applying such a conservative adjustment could have substantially increased the risk of Type II errors, potentially obscuring meaningful associations. Nonetheless, we acknowledged that this approach may increase the likelihood of false-positive findings; therefore, the results should be interpreted with appropriate caution. Third, the administration of the serial subtraction task during the cognitive dual-task walking condition was not standardized. While the use of randomly selected three-digit starting numbers helped maintain cognitive load across repeated trials, it may have introduced variability in task difficulty. This variability could have influenced participants' cognitive performance and, in turn, affected their gait performance. Fourth, this study lacked a secondary task performance assessment. Since dual-task interference may affect secondary task performance, exploring dual-task costs associated with secondary tasks is essential. Fifth, executive function subdomains have diverse definitions, with no consistent standard. The selection of commonly used and clinically feasible subdomains in this study may also be a limitation. Furthermore, the absence of detailed clinical profiling, especially regarding PD subtypes and cognitive reserve indicators beyond years of education, may serve as confounders and should be considered when interpreting the results.

This study was designed to investigate the associations between executive function subdomains and dual-task walking performance in PwPD, with the aim of identifying cognitive predictors of gait performance under dual-task conditions. As the focus was on within-group relationships specific to PwPD, a healthy control group was not included. However, future studies may consider including an age-matched healthy control group to provide comparative data, which could help further contextualize the findings and determine the extent to which these cognitive-motor associations are unique to PwPD or reflect broader aging-related processes.

## 5 Conclusion

This study found that executive function, especially inhibitory control, plays a key role in dual-task walking in PwPD. By recognizing the role of executive function, particularly inhibitory control, in gait regulation, clinicians can develop more effective interventions that combine cognitive and motor training to enhance mobility and prevent falls in individuals with PD.

## Data Availability

The datasets presented in this article are not readily available because the dataset is not publicly available due to ethical considerations. Access to the dataset may be granted upon reasonable request to the corresponding author, subject to institutional and ethical approvals. Requests to access the datasets should be directed to Yea-Ru Yang, yryang@nycu.edu.tw.
